# Editorial: Powering the next-generation IoT applications: new tools and emerging technologies for the development of Neuromorphic System of Systems

**DOI:** 10.3389/fnins.2023.1197918

**Published:** 2023-05-04

**Authors:** Gianvito Urgese, Antonio Rios-Navarro, Alejandro Linares-Barranco, Terrence C. Stewart, Konstantinos Michmizos

**Affiliations:** ^1^Politecnico di Torino, EDA Group, Torino, Italy; ^2^Robotics and Tech of Computers Group, SCORE Lab, ETSI-EPS, Sevilla, Spain; ^3^National Research Council Canada, Ottawa, ON, Canada; ^4^Computational Brain Lab, Department of Computer Science, Rutgers University, Piscataway, NJ, United States

**Keywords:** brain-inspired computational primitives, neuromorphic engineering, neuromorphic IoT applications, neuromorphic tools, sensory fusion, neuromorphic computing, neuromorphic framework

## 1. Introduction

The brain, this 3-pound mass of tissue that can easily be held in one's palm, has an inherent computational complexity that has always inspired efforts to endorse machines with some of its remarkable characteristics. Ironically, the brain computes in its own way, compared to analog or digital computers, despite sharing key concepts with both. It employs analog computation but digital communication, through spikes, both of which improve robustness to noise. This unique combination defines a new computational paradigm that we have just started to explore.

The reasons why neuromorphic systems are one of the fastest growing applications are not purely scientific, but mainly technological. For 50 years, the principle guiding computations has been Moore's Law, a macroscopic observation that we will always find ways to engineer faster, smaller and cheaper chips. But there are several reasons why Moore's Law is no longer able to keep up. First, physics: As we downsize transistors close to the atomic scale, it becomes difficult to regulate electron flow. Electrons do not necessarily adhere to Newtonian physics and may pass through the transistor barriers, a phenomenon called quantum tunneling. This makes our computer architectures *inefficient*. Second, we have long accepted the existence of a trade-off between computing faster and consuming less power, but this has never been a problem until we started approaching the physical limits of fabricating transistors. And the final nail in Moore's Law coffin is put by deep learning. Our computational needs are now orders of magnitude higher than what our systems can deliver.

The von-Neumann paradigm is already having more than its fair share of inefficiency—to match its brilliance. And the reason is simple. Computers have been designed with *feasibility*, not *efficiency*, at their center. And nowhere are the effects of a bad design more imminent—or the opportunity for an alternative design more compelling – than in emerging technologies, such as edge intelligence, where the computing needs become distilled, asking for real-time solutions to problems constrained by big data. A deep network running on a wearable device will deplete its battery within minutes. The sensors of an autonomous car can easily generate 1 GBps of data. These are examples of the need for real-time computing. The explosive growth of IoT is limited by the *efficiency* of our computing systems. We are nowhere near ready or prepared for this computational tsunami.

There is no better time than this to reconsider the feasibility of alternative solutions. What we need is a computing paradigm that is *versatile, robust* and *power efficient*, to handle these seismic shifts in our needs. And what we have now, is enough knowledge on how the brain can achieve these goals. The brain is fault tolerant. It is also extremely power efficient. And it becomes useless if you detach it from its environment: It performs self-learning, one of its most important attributes, by self-organizing based on the input it receives from the environment and other brains.

In this topic, we present efforts for advancing non-Von Neumann computations that draw from the brain's functional analogies. Below we walk through the rationale, the challenges and the advantages of redefining algorithms as spiking neural networks, where memory, learning and computing are tightly integrated to advance the implementation of enhanced IoT solutions.

## 2. Overview

Within IoT 2.0 and Industry 4.0 paradigms, the transition from cloud to edge computing is vital for homogeneous and universal data access throughout smart connected devices and product life cycles. This calls for more advanced edge devices, which often struggle with power constraints; one of the key players in this challenge is the neuromorphic technology, inspired by the most advanced and power-efficient sensor data analytic system—the human brain. Although initially intended for brain simulations, the adoption of the emerging neuromorphic technology is more and more appealing in fields such as IoT edge devices, Industry 4.0, biomedical, HPC, and robotics. This trend is confirmed by the effort of several companies in developing neuromorphic architectures and software tools that are opening the way to a new family of hybrid neuromorphic/digital IoT devices that will gain benefits from the novel neuro-inspired features such as stochasticity, low latency, structural plasticity, event-driven computation, and temporal sparse information coding.

Neuromorphic computational paradigms and hardware architectures are now mature enough to play an important role in IoT applications running on the edge, because of their ability to learn and adapt to ever-changing conditions and tasks while respecting limited power requirements. Several state-of-the-art benchmark applications have proved that Neuromorphic solutions, since brain-inspired, provide better scalability than traditional multi-core architectures, and are especially suited for low-power and adaptive applications required to analyze data in real-time.

However, the weak standardization of Neuromorphic components, tools, and frameworks makes it challenging to define the engineering process for developing and orchestrating hybridized Neuromorphic/Digital System of Systems deployable in real-world application scenarios. Our Research Topic focus on various theoretical and practical aspects of different Neuromorphic setups for facilitating the adoption of Neuromorphic technology into the design of a System of Systems products and algorithms for IoT applications. Papers in this Research Topic describe the latest advances in research on neuromorphic computational paradigms, encoding algorithms, frameworks, toolchains, tools and applications which will act as a reference for application developers involved in the design of hybrid digital/neuromorphic systems for the IoT domain.

Putra et al. proposed EnforceSNN a novel design framework which enables the design of resilient and energy-efficient SNNs considering approximate DRAMs for embedded systems while minimizing their negative impact on the accuracy of the application. The framework provides a solution for resilient and energy-efficient SNN inference using reduced-voltage DRAM for embedded systems.

Kleijnen et al. report the capability of a network simulator to provide representative results of the network load measured on the SpiNNaker board running a set of benchmark SNNs. Thus, providing a powerful tool to be adopted in the early phases of the solution design.

 Müller-Cleve et al. report on the detailed implementation of a benchmark for spatiotemporal tactile pattern recognition at the edge. The authors integrated a full pipeline for implementing the Braille letter reading task using neuromorphic and digital tools and architectures. Then, the use case has been analyzed to highlight the strengths and weaknesses of the neuromorphic solution against the pure digital version.

Forno et al. analyzed the signal-to-spike encoding techniques with an information theory-based approach by evaluating metrics like entropy, mutual information, efficiency, sparsity, and coding efficiency. The analysis has been performed on the most common spike encoding algorithms using audio and IMU signals as sources. Thus, providing reference indications for system designers during the engineering of the solution.

Nilsson e al. report on the current landscape of neuromorphic computing, focusing on characteristics that pose integration challenges between digital and neuromorphic technologies. Based on this analysis, the authors proposed a microservice-based conceptual framework for neuromorphic systems integration.

## Author contributions

All authors listed have made a substantial, direct, and intellectual contribution to the work and approved it for publication.

